# Anthropometric Indices of Obstructive Sleep Apnea Patients in Mauritius

**DOI:** 10.7759/cureus.72708

**Published:** 2024-10-30

**Authors:** Vibhootee Sant Bakshsingh, Meera Manraj, Devaraj Keser-I-Hind Pillai, Fateemah Suhootoorah, Abdool Arbaaz Boodhun, Sidrah Bibi Soreefan

**Affiliations:** 1 Faculty of Medicine, University of Mauritius, Reduit, MUS

**Keywords:** anthropometric indices, apnea-hypopnea index, bmi, neck circumference, neck circumference-to-height ratio, obstructive sleep apnea (osa), waist circumference, weight circumference-to-height ratio

## Abstract

Background

Obstructive sleep apnea (OSA) is the most prevalent sleep-related breathing disorder, affecting a significant number of individuals globally. It is associated with poor quality of life and an increased risk of all-cause mortality. It is estimated that most of the patients suffering from OSA are obese. Anthropometric indices can help guide doctors toward the diagnosis.

Objectives

This study aimed to establish specific anthropometric thresholds associated with OSA risk, allowing clinicians to identify individuals more likely to have OSA and who may need further evaluation, such as polysomnography. This targeted screening approach would enable better resource allocation to those at higher risk, potentially enhancing the efficiency of OSA diagnosis and management.

Methods

We conducted a descriptive study using data from the sole private sleep clinic in Mauritius. We analyzed case files of consecutive patients diagnosed with OSA over a 3.5-year period from January 2015 to June 2018.

Results

The study population comprised 170 patients (79.4% males) diagnosed with OSA by a polygraphy test. The mean age of OSA diagnosis in men and women was 51.7 ± 14.0 years and 53.8 ± 12.5 years, respectively. The mean neck circumference (NC) for males and females was 41.9 ± 3.9 cm and 38.2 ± 3.9 cm, while the mean waist circumference (WC) was 105.6 ± 12.2 cm for men and 103.4 ± 14.5 cm for women. The body mass index (BMI) was 29.1 ± 5.0 kg/m^2^ and 31.4 ± 6.8 kg/m^2^ for males and females, respectively. The apnea-hypopnea index (AHI) averaged to 42.1 ± 19.7 events/hour for men and 33.3 ± 16.4 events/hour for women. In male patients, we found a positive significant correlation (p<0.001) between AHI and the following parameters: BMI (r=0.443), WC (r=0.337), WC-to-height ratio (WHr) (r=0.378), NC (r =0.274), and neck-to-height ratio (NHr) (r =0.321). In women, we observed a positive significant correlation between apnea severity and the following: BMI (r=0.396, p=0.029), WC (r=0.462, p=0.005), and WHr (r=0.494, p=0.003). No significant relationships were observed between AHI and the following parameters in women: NC (r=0.317, p=0.064) and NHr (r=0.311, p=0.069). A total of 83.5% of patients had a Mallampati score of 3-4.

Conclusion

This study represents a pioneering effort on the island. While further research is necessary to establish exact anthropometric cutoff values, the findings offer crucial insights for physicians to identify high-risk individuals. With just a scale, measuring tape, and calculator, healthcare professionals can detect important health markers that extend beyond diagnosing OSA. These simple measurements not only help in predicting OSA but also provide a broader view of an individual's overall health, identifying risks that go beyond sleep issues. This research sets an important foundation for future OSA studies within the Mauritian population.

## Introduction

Issador Ducasse Lautreamont once described sleep as a *reward for some, a punishment for others.* This dichotomy mirrors the experience of individuals with obstructive sleep apnea (OSA), a pervasive yet often unrecognized chronic respiratory disorder. Patients with OSA experience episodes of reduced (hypopnea) or absent (apnea) airflow due to recurrent airway obstruction, characterized by loud snoring, episodes of hypoxemia, and apnea, which are typically terminated by brief arousals resulting in sleep fragmentation and unrefreshed sleep [1].

The global impact of OSA is substantial, underscoring its relevance as a public health concern. Approximately one billion people worldwide are affected by OSA, with 936 million adults aged 30-69. Of these, approximately 425 million are believed to have moderate to severe OSA [2]. This high prevalence underscores the widespread nature of the disorder and the need for increased attention and resources dedicated to its management and treatment.

OSA is independently associated with an increased risk of all-cause mortality [3]. Despite its high prevalence and serious consequences, including hypertension, stroke, and coronary artery disease, OSA remains underdiagnosed [4]. Obesity is the strongest risk factor, affecting 60% of patients with OSA [5].

The Sleep Heart Health Study asserts that body mass index (BMI), neck circumference (NC), and waist circumference (WC) independently pose risks for developing OSA [6]. Davidson and Patel's research [7] further strengthens this connection, demonstrating that OSA correlates more strongly with increased NC and WC than BMI. Despite the lack of established standard cutoffs, the values commonly utilized to suspect OSA include WC > 90 cm for males and > 80 cm for females, alongside NC > 43 cm for men and > 38 cm for women [8].

Reflecting the global challenge of OSA, the situation in Mauritius presents a microcosmic view of this pressing health issue, with obesity serving as a harbinger for the prevalence of OSA. With the 2021 Non-Communicable Disease (NCD) report [9] indicating that over 70% of the population of Mauritius possesses a high BMI, the potential scale of OSA among the population becomes alarming. This alarming health trend was previously highlighted in Mauritius's 2009 NCD report [10], revealing a significant risk of sleep apnea within the Mauritian population, with a notable prevalence of 21.4% (24.1% among women and 18.7% among men). Given the escalating rates of obesity on the island, it is anticipated that this figure has substantially increased since then.

Our research aims to establish specific anthropometric benchmarks tailored to the unique demographic characteristics of Mauritians. In a setting where healthcare resources are limited, these anthropometric thresholds will be essential for clinicians to effectively assess potential OSA cases and initiate early management strategies.

## Materials and methods

A retrospective descriptive study was conducted following ethical approval from the Department of Medicine Research and Ethics Committee of the University of Mauritius.

The study population comprised all consecutive patients diagnosed with OSA over a 3.5-year period from January 2015 to June 2018. Data were extracted from clinical records at the island's sole private sleep apnea clinic. A total of 207 patients diagnosed with OSA were initially identified. After excluding 18% of case files due to incomplete or missing essential information, 170 case files were included in the final analysis. All collected data were anonymized to ensure patient confidentiality by assigning unique code numbers.

Patients suspected of having sleep apnea underwent polygraphy (PG) testing using a standard Home Sleep Apnea Testing (HSAT) Type 3 device (SOMNOlab 2, Weinmann, Hamburg, Germany). Those with ambiguous results underwent a polysomnography test. All patients included in the study were diagnosed with OSA using the PG test. The inclusion criteria were defined as age ≥18 years and an apnea-hypopnea index (AHI) of ≥5 events per hour. The exclusion criteria encompassed non-Mauritian nationals and pregnant women.

The following parameters were extracted from the patient's medical records: gender, age at OSA diagnosis, height, weight, WC (midpoint between the lower border of the rib cage and the iliac crest in the upright position, just above the umbilicus), NC (measured at the level of the cricothyroid membrane), modified Mallampati score (MMS), and AHI. BMI, WC-to-height ratio (WHr), and NC-to-height ratio (NHr) were calculated from the recorded anthropometric data.

All statistical analyses were performed using IBM SPSS Statistics for Windows, Version 22 (Released 2013; IBM Corp., Armonk, New York). Continuous variables were presented as mean ± standard deviation, while categorical variables were expressed as numbers and percentages, n (%). The Student's t-test was used to evaluate the statistical significance of continuous variables, and the chi-square test was used for categorical data. Pearson's correlation test was used to assess the relationships between various variables and OSA severity, as measured by AHI. A p-value of less than 0.05 was considered statistically significant.

## Results

A total of 170 individuals participated in this study, comprising 135 men (79.4%) and 35 women (20.6%). Table 1 details the characteristics of the study population. Table 2 presents the relationship between the severity of OSA (measured by AHI) and the various variables assessed.

**Table 1:** Characteristics of the study population Data are presented as mean ± standard deviation. The Student's t-test was used to assess statistical significance regarding gender differences in the measured variables. A p-value of <0.05 was considered significant.

Parameters	Males (n=135)	Female (n=35)	P-value	Total Study Population (n=170)
Age, years	51.7 ± 14.0	53.8 ± 12.5	0.430	52.2 ± 13.7
Height, m	1.73 ± 0.07	1.60 ± 0.06	<0.001	1.71 ± 0.09
Weight, kg	88.0 ± 15.6	83.0 ± 20.8	0.043	86.5 ± 16.9
BMI, kg/m^2^	29.1 ± 5.0	31.4 ± 6.8	0.041	30.6 ± 5.4
Waist circumference (WC), cm	105.6 ± 12.2	103.4 ± 14.5	0.368	105.1 ± 12.7
Waist circumference-to-height ratio (WHr), cm/m	0.61 ± 0.07	0.65 ± 0.09	0.004	0.62 ± 0.08
Neck circumference (NC), cm	41.9 ± 3.9	38.2 ± 3.9	<0.001	41.1 ± 4.2
Neck circumference-to-height ratio (NHr), cm/m	24.2 ± 2.4	23.9 ± 2.6	0.517	24.1 ± 2.49
Apnea-hypopnea index (AHI), events/hour	42.1 ± 19.7	33.3 ± 16.4	0.017	40.3 ± 19.4

**Table 2:** Correlations between the different parameters studied and OSA severity The table summarizes the correlation between the various parameters studied and OSA severity. A Pearson correlation test was used to assess these relationships.

AHI and Different Variables	Pearson Correlation (Males)	P-value (Males)	Pearson Correlation (Females)	P-value (Females)	Pearson Correlation (Total Population)	P-value (Total Population)
BMI	0.443	< 0.001	0.396	0.029	0.376	< 0.001
Neck circumference (NC)	0.274	0.001	0.317	0.064	0.323	< 0.001
Waist circumference (WC)	0.337	< 0.001	0.462	0.005	0.364	< 0.001
Neck circumference-to-height ratio (NHr)	0.321	< 0.001	0.311	0.069	0.320	< 0.001
Waist circumference-to-height ratio (WHr)	0.378	< 0.001	0.494	0.003	0.347	< 0.001

BMI

A total of 90% of patients had a BMI above normal limits (≥ 25 kg/m^2^). The difference in BMI between men and women was statistically significant, with men having a mean BMI of 29.1 ± 5.0 kg/m^2^ and women 31.4 ± 6.8 kg/m^2^ (p-value = 0.041).

**Figure 1:**
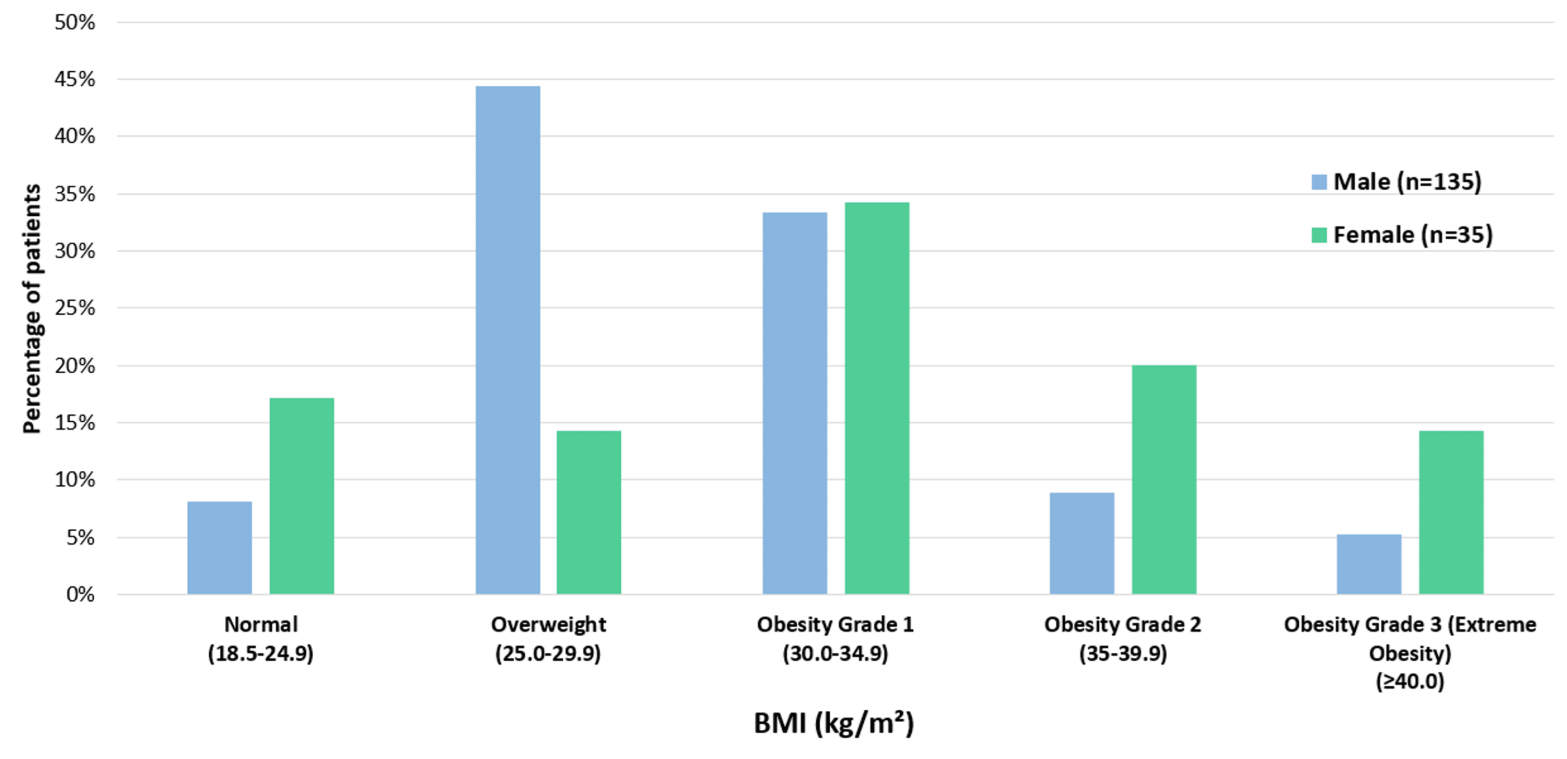
Distribution of patients by obesity severity based on WHO classification The bar chart shows the distribution of male and female patients across various BMI categories. Among women, six (17.1%) had a BMI in the normal range compared to 11 (8.2%) of men. A significant portion of men, 60 (44.4%), were classified as overweight, while only five (14.3%) women fell into this category. In the Obesity Grade 1 category, there were 45 (33.3%) men and 12 (34.3%) women. For Grade 2 obesity, 12 (8.9%) men and seven (20.0%) women were represented. Lastly, in the Grade 3 (extreme obesity) category, seven (5.2%) men and five (14.3%) women were included.

We observed a higher severity of apnea among individuals with greater BMI. A Pearson correlation analysis revealed a strong positive relationship between BMI and AHI in males (r = 0.443, p < 0.001), with a similarly significant correlation in females (r = 0.396, p = 0.029). Figure 1 illustrates the distribution of patients according to the WHO's obesity classification [11].

WC

The mean WC for men and women in the study was 105.6 ± 12.2 cm and 103.4 ± 14.5 cm, respectively, with no statistically significant difference between the sexes (p = 0.368). According to ethnic-specific cutoffs set by the WHO for minimal health risk related to WC, thresholds of < 90 cm for men and < 80 cm for women were used [11]. Among the participants, 126 out of 135 males had a WC of ≥ 90 cm, and 34 out of 35 females had a WC of ≥ 80 cm.

Larger WC measurements were significantly associated with increased OSA severity. Pearson correlation analysis revealed a significant positive relationship between WC and apnea severity in both males (r = 0.337, p < 0.001) and females (r = 0.462, p = 0.005).

WHr

The mean WHr for men and women in the study was 0.61 ± 0.07 and 0.65 ± 0.09, respectively. The difference in mean WHr between men and women was statistically significant (p = 0.004). According to Gibson and Ashwell, WHr is considered a better indicator of health risk than other measures, with a cutoff value of 0.5 being a recognized marker for cardio-metabolic risk [12]. In our study, 163 out of 170 patients (95.9%) had a WHr ≥ 0.5.

Pearson correlation analysis demonstrated a significant positive relationship between apnea severity and WHr in both genders: males (r = 0.378, p < 0.001) and females (r = 0.494, p = 0.003).

NC

Currently, no standard classification exists for NC. In this study, the mean NC for males was 41.9 ± 3.9 cm and 38.2 ± 3.9 cm for females. The difference in mean NC between males and females was statistically significant (p < 0.001). Pearson correlation test revealed a significant positive correlation between NC and apnea severity in males (r = 0.274, p = 0.001). However, no significant correlation was found in females (r = 0.317, p = 0.064).

NHr

NHr was calculated for the study population, with mean values of 24.2 ± 2.4 cm/m for males and 23.9 ± 2.6 cm/m for females. According to Ho et al., an NHr >25 cm/m is associated with a higher likelihood of OSA [13]. In our study, 50 participants had an NHr above 25 cm/m, including 40 males (29.6%) and 10 females (28.5%). Additionally, 75% of the study population had an NHr ≥ 22 cm/m. Pearson correlation analysis revealed a significant positive relationship between NHr and apnea severity in males (r = 0.321, p < 0.001), while no significant correlation was observed in females (r = 0.311, p = 0.069).

MMS

Out of 170 patients, 118 males and 27 females, accounting for 85.3% of the total cohort, exhibited an MMS of 3-4. In males, the mean AHI for MMS of 1-2 was 37.6 ± 18.9 events/hour, compared to 42.8 ± 19.8 events/hour for scores 3-4, with no statistically significant difference observed (p = 0.315). In females, the mean AHI for scores 1-2 was 30.8 ± 18.4 events/hour, while for scores 3-4, it was 34.1 ± 16.1 events/hour, also demonstrating no statistically significant difference (p = 0.618).

AHI

**Figure 2:**
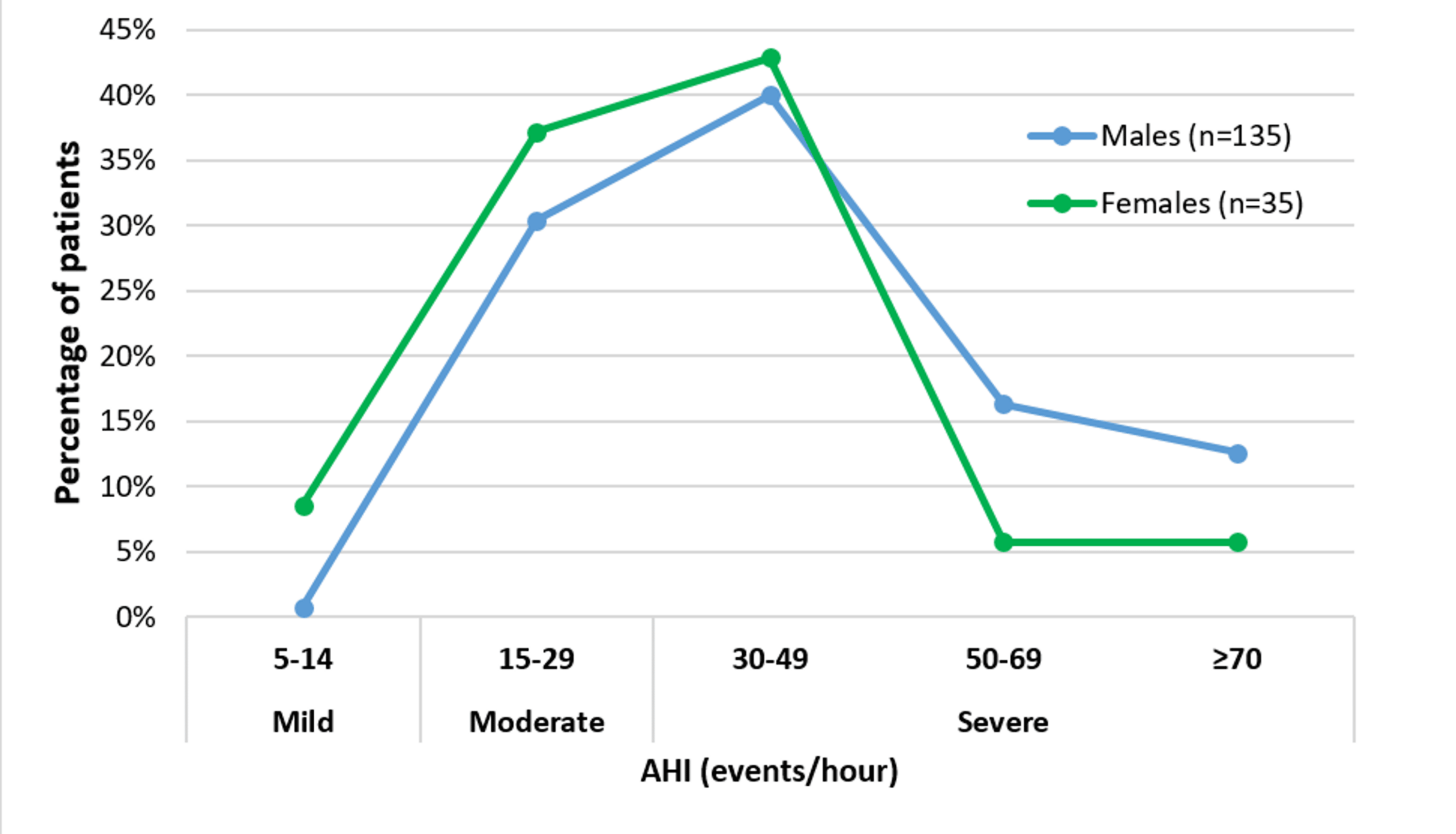
Distribution of patients according to OSA severity The line graph illustrates the distribution of patients based on the severity of their obstructive sleep apnea (OSA). For mild OSA, the prevalence is notably higher in females, with three (8.9%) compared to one (0.7%) in males. In moderate OSA cases, females also outnumber males, with 13 (37.1%) compared to 41 (30.4%) males. However, in cases of severe OSA (AHI ≥ 30), males are more represented, with 93 (68.5%) compared to 19 (54.2%) females.

The overall mean AHI was 40.3 ± 19.4 events/hour for our study population. The average AHI for men was 42.1 ± 19.4 events/hour, while for women, it was 33.3 ± 16.4 events/hour. The difference in mean AHI between genders was statistically significant (p = 0.017). Figure 2 illustrates the distribution of OSA severity within our study population.

## Discussion

The average AHI observed in our study population was 40.3 events per hour, with 68.5% of males and 54.2% of females exhibiting severe OSA, a concerning figure.

OSA has long been labeled a "disease of fat people*.*" While this is partly true, as obesity is the most recognized risk factor for OSA, occurring in over 60% of cases [5], the relationship between OSA and obesity is more nuanced. Sullivan and Issa, over three decades ago, argued that although obesity often correlates with more severe OSA, it is not a definitive marker for the condition [1]. Our study supports this view, as nearly half (48.2%) of the participants had a BMI below 30 kg/m^2^, with a notable 10% within the normal BMI range. Therefore, the absence of obesity does not exclude the possibility of OSA, contrary to popular belief.

A Pearson correlation coefficient revealed a significant positive correlation between the severity of apnea and BMI, reinforcing Sullivan and Issa's earlier hypothesis [1]. In Mauritius, the obesity challenge is clear, with 36% of the population classified as overweight and 36.2% as obese [9]. Our research found that 90% of the study population had BMIs above normal limits. Vgontzas et al. [14] identified OSA in approximately 41% of individuals with a BMI greater than 28 kg/m^2^, suggesting that OSA may represent a silent epidemic affecting a substantial portion of the Mauritian community.

While the strong link between OSA and BMI is evident, the predictive value of BMI is being reconsidered. Increasing evidence suggests that NC and WC might be better indicators of OSA risk, as they more accurately measure central body fat than BMI, which gauges overall body fat. Deng et al. proposed that NC and WC could independently predict OSA risk [15]. This evolving perspective highlights the importance of body fat distribution over general body mass when assessing OSA risk.

NC is a marker of upper body fat distribution. Our findings indicate that, on average, males had an NC of 41.9 cm, while females measured 38.2 cm. Current guidelines suggest that an NC exceeding 40 cm poses a risk for OSA [16]. However, these thresholds are predominantly based on data from Caucasian populations and may not fully apply to other ethnic groups, such as the Mauritian population. Ardelean et al. [17] identified a 41 cm threshold as predictive of OSA in 95.9% of cases among a sample of 836 males, although this observation was limited to a Caucasian context, raising questions about its broader applicability.

Based on our study, which found that 75% of males and females had an NC of 40 cm and 38 cm or greater, respectively, we recommend revising the NC threshold. We suggest that individuals with these measurements or higher should be considered at high risk of developing OSA. Our study also found a significant positive correlation between apnea severity and NC in men.

WC is regarded as a more accurate measure of central adiposity than BMI and a better predictor of all-cause mortality [18]. In our patient population, the mean WC was 105.6 cm for men and 103.4 cm for women. A Korean study of 382 OSA patients found that a WC greater than 76.5 cm for women and 88.5 cm for men could predict OSA with a sensitivity of 88% and specificity of 54% for women, and a sensitivity of 81% and specificity of 64% for men [19]. However, these cutoff values may differ for our population.

Using ethnic-specific cutoff values from the WHO, a large waist is defined as WC ≥ 90 cm for males and ≥ 80 cm for females. In our study population, 93.3% of men and 97.1% of women had WC measurements above these thresholds. According to the latest NCD report [9], 62.8% of the Mauritian population (54.7% of men and 69.9% of women) have a large waist. This suggests that physicians should consider screening for OSA in patients with large waists. Our study showed a positive Pearson correlation between apnea severity and WC in both males and females.

Ho et al. [12] found that the NHr serves as a superior predictor of OSA. Their research demonstrated that an NHr exceeding 25 cm/m was associated with an 18-fold increase in OSA risk, with a positive predictive value of 25% and a negative predictive value of 96%. In our study, only 30% of participants had an NHr exceeding 25 cm/m, although 75% had an NHr of 22 cm/m or more. This variation suggests that the NHr cutoff may need to be adjusted for our demographic. We propose using a lower NHr threshold of 22 cm/m for screening OSA within our population.

Over a decade ago, it was suggested that WC should ideally not exceed half of an individual's height [20]. The WHr, with a threshold of 0.5 or greater, was found to correlate more strongly with all-cause mortality than other obesity metrics [21]. While a definitive WHr cutoff for OSA screening has yet to be established, the clear link between central adiposity and OSA suggests that individuals with a WHr of 0.5 or above should be screened for OSA. Our study supports this, with 95.9% of participants presenting a WHr of 0.5 or greater.

Furthermore, over 85% of our study population had an MMS of 3-4. According to Nuckton et al. [22], the Mallampati score is an independent risk factor for OSA, with each one-point increase doubling the likelihood of developing the condition. This underscores the importance of the Mallampati score as a simple yet valuable tool for screening OSA.

This study is the first to examine anthropometric indices among OSA patients on the island. The sample size suggests that the results are statistically significant. Additionally, the physical examinations and anthropometric measurements were conducted by the same doctor and nursing staff, minimizing potential inter-observer bias. The findings suggest that analyzing anthropometric indices can offer valuable insights for presumptive OSA diagnosis, with proposed cutoff values that can be easily implemented in clinical practice.

Limitations

This study provides valuable insights into OSA in the Mauritian population, but several limitations must be acknowledged. Firstly, the study's sample, drawn from a private clinic, may not fully represent the broader Mauritian population due to potential socioeconomic biases. Additionally, the gender imbalance, with 79.4% male participants, reflects typical sleep clinic referral patterns but limits the generalizability of the findings across genders. This skewed distribution may affect the study's applicability to the overall population. Future research should include a more balanced gender representation and a larger sample size to offer comprehensive insights into OSA prevalence in Mauritius.

Furthermore, the use of polygraphy for OSA diagnosis instead of the gold standard polysomnography might have led to an underestimation of AHI severity, as apnea events were measured based on recording time rather than actual sleep time. The absence of a non-OSA control group presents another limitation, as it restricts the study's ability to establish definitive anthropometric cutoff values and develop robust screening thresholds for OSA. This lack of a comparative baseline may reduce the accuracy in distinguishing OSA-specific anthropometric indices from those in the general population. Despite these limitations, the findings offer a foundational framework for further studies and can help clinicians in identifying individuals at risk for OSA. However, future studies should prioritize the inclusion of a well-defined non-OSA control group to improve the validity of the results and allow for a more precise determination of OSA-specific anthropometric thresholds.

Lastly, the cross-sectional nature of the study only provides a snapshot of the relationship between anthropometric indices and OSA, limiting the ability to establish causality or observe temporal changes in these indices relative to OSA progression or treatment. Future research should focus on larger, more diverse samples with balanced gender representation, as well as longitudinal studies, to address these limitations and enhance our understanding of OSA in various populations.

## Conclusions

While further research is needed to establish precise anthropometric cutoff values, the findings offer valuable guidance for physicians in identifying high-risk individuals. Using a weighing scale, measuring tape, and a calculator, healthcare practitioners can uncover critical health indicators that extend beyond OSA diagnosis. These simple anthropometric indices not only help predict OSA but also provide insights into an individual's overall health, revealing risks that go beyond disrupted sleep. This study lays an important foundation for future research on OSA within the Mauritian population.
